# Increased Generation of HIV-1 gp120-Reactive CD8^+^ T Cells by a DNA Vaccine Construct Encoding the Chemokine CCL3

**DOI:** 10.1371/journal.pone.0104814

**Published:** 2014-08-14

**Authors:** Inger Øynebråten, Jorma Hinkula, Agnete B. Fredriksen, Bjarne Bogen

**Affiliations:** 1 Dept. of Immunology, University of Oslo and Oslo University Hospital – Rikshospitalet, Oslo, Norway; 2 Centre for Immune Regulation, University of Oslo, Oslo, Norway; 3 Division of Molecular Virology, Dept. of Clinical and Experimental Medicine, Linköping University, Linköping, Sweden; 4 KG Jebsen Centre for research on Influenza Vaccines, University of Oslo, Oslo, Norway; Federal University of São Paulo, Brazil

## Abstract

DNA vaccines based on subunits from pathogens have several advantages over other vaccine strategies. DNA vaccines can easily be modified, they show good safety profiles, are stable and inexpensive to produce, and the immune response can be focused to the antigen of interest. However, the immunogenicity of DNA vaccines which is generally quite low needs to be improved. Electroporation and co-delivery of genetically encoded immune adjuvants are two strategies aiming at increasing the efficacy of DNA vaccines. Here, we have examined whether targeting to antigen-presenting cells (APC) could increase the immune response to surface envelope glycoprotein (Env) gp120 from Human Immunodeficiency Virus type 1 (HIV-1). To target APC, we utilized a homodimeric vaccine format denoted vaccibody, which enables covalent fusion of gp120 to molecules that can target APC. Two molecules were tested for their efficiency as targeting units: the antibody-derived single chain Fragment variable (scFv) specific for the major histocompatilibility complex (MHC) class II I-E molecules, and the CC chemokine ligand 3 (CCL3). The vaccines were delivered as DNA into muscle of mice with or without electroporation. Targeting of gp120 to MHC class II molecules induced antibodies that neutralized HIV-1 and that persisted for more than a year after one single immunization with electroporation. Targeting by CCL3 significantly increased the number of HIV-1 gp120-reactive CD8^+^ T cells compared to non-targeted vaccines and gp120 delivered alone in the absence of electroporation. The data suggest that chemokines are promising molecular adjuvants because small amounts can attract immune cells and promote immune responses without advanced equipment such as electroporation.

## Introduction

Vaccines based on live attenuated pathogen often elicit strong, lifelong immune responses and protection against disease. However, safety and efficacy in immunocompromised individuals is a concern. In addition, live attenuated pathogens have the very rare potential to revert to a pathogenic form [1]. Therefore, alternative vaccine strategies are desired. Killed or inactivated pathogens may be used, but side effects may still be a problem as well as lower efficacy. A promising alternative utilizes pathogen-derived subunits delivered as protein or DNA. Subunit-based vaccines show good safety, and in particular DNA vaccines are easy and fast to produce and are stable in terms of storage and temperature changes [2], [3]. Three successful DNA vaccines have been licensed for animal use [4], [5], [6], and several clinical trials with DNA vaccines have been conducted in humans [2] (clinicaltrials.gov).

Whereas pathogens harbour potent immunostimulatory molecules, these are often lost in the subunit-based vaccines. Thus, in recent years, several attempts have been made aiming at increasing the immunogenicity of such vaccines [3], [7], [8]. For subunit-based DNA vaccines, two improvements include *in vivo* electroporation and delivery of genetically encoded immune adjuvants. Electroporation can enhance cellular uptake of DNA, increase DNA distribution throughout the tissue, and cause a local inflammatory reaction. All these events contribute to a stronger immune response [7],[9]. The two most widely tested immune adjuvants are the cytokines granulocyte macrophage colony-stimulating factor (GM-CSF) and interleukin (IL)-12, which both can improve immune response [7], [10], [11], [12]. To further improve the response, electroporation may be combined with delivery of adjuvants [7], [8].

Targeting of antigen to endocytic molecules present on antigen-presenting cells (APCs) is another strategy that is utilized to increase immunogenicity of pathogen-derived subunits delivered as protein or as DNA. This can improve effectiveness of vaccines, reduce the amount of antigen needed, and it may also promote cross-presentation of antigens [13], [14], [15], [16]. Several targeting approaches utilize the ligand-binding properties of the variable regions of antibodies, and already in the 80s, targeting by an antibody specific for immunoglobulin or major histocompatibility complex (MHC) class II was utilized to increase immune responses [17], [18]. A widely tested targeting approach for HIV-1-derived subunits is their fusion to the C-terminus of an antibody specific for the type I C-type lectin DEC205 (CD205). Upon co-delivery with a toll-like receptor (TLR) 3-agonist, this approach results in increased antigen-specific CD4^+^ and CD8^+^ T cell responses [19], [20], [21]. Finally, chemokines may be utilized [22], [23], [24], [25], [26], [27], [28], [29], [30]. Chemokines can recruit APCs expressing the corresponding chemokine receptors to the injection site of the vaccine, and promote cellular uptake of the vaccine antigen into endocytic compartments of APCs. One example is the chemokine CCL3 which is a ligand of the chemokine receptors CCR1 and CCR5 and which production is inducible in numerous cell types including cells of the immune system, epithelial cells, and fibroblasts [31], [32]. In mouse models, CCL3 has been shown to recruit Langerhans cells, dendritic cells (DCs), as well as monocyte derived DCs, suggesting that these cells express either CCR1 or CCR5 or both [33], [34], [35]. CCR1 and CCR5 are also expressed by macrophages, NK cells, CD4^+^ and CD8^+^ T cells [36], [37].

In this study, we have examined whether DNA vaccines that target HIV-1 surface envelope glycoprotein (Env) gp120 to APC, could increase the immune responses towards the antigen. To protect against HIV-1 and development of AIDS, antibodies that can neutralize a broad number of virus variants, and CD8^+^ T cells that can eradicate virus-infected cells, are likely to be important [38], [39]. In previous studies, targeting of tumor antigens or the influenza virus antigen hemagglutinin to APC MHC class II molecules, were shown to increase the antibody responses compared to non-targeted delivery [27], [40], [41]. Moreover, targeting by the chemokine CCL3 induced CD8^+^ T cells that were important for protection against tumor development and influenza-mediated disease in mouse models [24], [41]. Thus, in order to examine whether we could promote antibody responses as well as CD8^+^ T cell responses towards an HIV-1 antigen, we utilized anti-MHC class II and CCL3 as targeting units. For this purpose we utilized a homodimeric vaccine format denoted vaccibody, which appears to have considerable flexibility regarding expression of targeting as well as antigenic units [22], [24], [26], [27], [40], [41], [42], [43]. The DNA vaccines were delivered intramuscularly to mice with or without electroporation. Targeting to MHC class II did not increase the antibody responses, however, one single immunization induced long-lasting antibodies that could neutralize HIV-1 when electroporation was included. In the absence of electroporation, targeting by CCL3 increased the number of HIV-1 gp120-reactive CD8^+^ T cells. The data are consistent with previous reports showing that CCL3 can increase CD8^+^ T cell immune responses [33], [41]. The ability to immunize without the need of advanced electroporation equipment and less pain, make CCL3 fusion vaccines an attractive approach in future DNA vaccine development.

## Materials and Methods

### Reagents

Recombinant gp120IIIB (produced in the baculovirus expression system) and V3-specific anti-gp120IIIB antibodies utilized in ELISA were purchased from ImmunoDX (Woburn, MA) or were together with gp120-spanning peptides (20 mers with 10 amino acid overlap), obtained from the Programme EVA Centre for AIDS Reagents, NIBSC, UK. The Programme EVA Centre for AIDS Reagents is supported by the EC FP6/7 Europrise Network of Excellence, AVIP and NGIN consortia, and the Bill and Melinda Gates GHRC-CAVD Project. Unless denoted, other reagents were from Sigma-Aldrich (St. Louis, MO).

### Mice

BALB/c mice were purchased from Taconic (Ry, Denmark) and were 6 to 12 weeks of age when included in the experiments. Before vaccination, the mice were anesthetized by s.c. injection of 200 µl of a mixture of Hypnorm (Fentanyl, 79 µg/ml; Fluanison, 2.5 mg/ml) and Dormicum (Midazolam 1.25 mg/ml). The experiments were performed at the Dept. of Comparative Medicine, Oslo University Hospital, Rikshospitalet. The study was approved by the National Committee for Animal Experiments (Oslo, Norway) (Permit number: Id-622, 2007 and Id-1400, 2010).

### Construction of vaccines

Construction of the vaccines containing Ig-derived hinge 1, hinge 4, and C_H_3 exons from human γ3 in the dimerization unit, and CCL3, CCL3C11S, anti-MHC class II, or anti-NIP in the targeting unit have previously been described [24], [40]. The HIV-1-derived gp120 gene was cloned from the plasmid VRC-gp120IIIB using specific primers including the SfiI restriction enzyme site in the 5′ and 3′ end of the gene. (Primers: SfiI restriction enzyme sites are underlined and the stop codon is indicated in bold. The gp120 codons are depicted in capital letters. 5′gp120: ta ggcctcagcggcctg AGT GCT ACA GAA AAA TTG TGG GTC ACA GT. 3′gp120: ta ggccctgcaggcc tca TCT TTT TTC TCT CTG CAC CAC TC). The PCR product of gp120 was inserted into the pLNOH2 vector [44], in the antigenic unit of the vaccine format.

### Characterization of the vaccine constructs

HEK293 cells were transfected with plasmids encoding the vaccine constructs by use of Lipofectamine 2000 according to the manufacturer’s instruction (Invitrogen, Life Technologies Co., CA). The cells were cultivated in RPMI 1640 with 10% fetal calf serum (FCS) or serum free media (FreeStyle 293, Gibco, Life Technologies) and the supernatants were harvested 3 to 6 days after transfection for analyses by ELISA, SDS-PAGE and Western blotting, receptor binding, and chemotaxis. Before SDS-PAGE and receptor binding experiments, the supernatants were subjected to Ultrafree spin columns (cutoff 50 kDa) (Millipore Co., MA) in order to concentrate the vaccine proteins.

#### ELISA

65 µl/well of capture antibodies (see table 1) diluted in PBS were incubated overnight in 96 well plates, then washed before blocking by use of 1% albumin (Biotest, Dreieich, Germany) in PBS for 1–2 hrs. All steps were performed at room temperature, and the plates were washed following each incubation step four times in PBS with 0.2% Tween-20. Samples, standards, and secondary reagents were diluted in ELISA-buffer (PBS containing 0.2% Tween-20, 0.1% albumin, and 0.02% sodium azide). Samples and standards in duplicates (50 µl/well) were incubated overnight followed by 75 µl/well of detection antibodies (see table 1) (incubation time 1.5–3 hrs). Next, alkaline phosphatase-conjugated streptavidin (1∶3000, GE Healthcare, UK) was added (incubation time minimum 1–2 hrs). P-nitrophenyl phosphate in diethanolamine buffer was added and developed for 10 to 60 minutes and the absorbance was measured at 405 nm with a Tecan Sunrise Microplate Reader (Tecan, Männedorf, Switzerland).

**Table 1:** Antibodies utilized in ELISA, Western blotting, and immunostaining.

Specificity	Clone/designation	Working concentration	Specification	Source
mCCL3	MAB450	4 µg/ml	Rat IgG2a	R&D Systems
mCCL3	BAF450	0.25 µg/ml	Biotinylated goat IgG	R&D Systems
αNIP	NIP-BSA	1 µg/ml		Rikshospitalet
gp120	Ab53937	1 µg/ml	Biotinylated goat IgG	Abcam
hFc	HP6017	1 µg/ml	Biotinylated mIgG2a	Sigma
hCH3	A57H	2 µg/ml	mIgM	AbD Serotec
mIgG Fc	A2429	1∶5000	AP-conjugated goat antibody	Sigma

m, mouse; h, human; AP, alkaline phosphatase.

#### Western blotting

In order to disrupt disulfide bonds, samples were treated with β-mercaptoethanol for 3 min at 95°C before electrophoresis. All samples were diluted in SDS-containing buffer and loaded on to 4–20% Novex Tris-Glycine polyacrylamide gel (Life Technologies). SeeBlue Plus2 Pre-Stained Standard ladder (Life Technologies) was used to indicate molecular weights (Mw). Following electrophoresis, the proteins were blotted onto a polyvinylidene difluoride membrane (Biorad, Hercules, CA) by 100 V for 1 h at 4°C. Milk and casein (5% and 1%, respectively) in 0.1% Tween-20 was used to block the membrane (1–2 hrs at room temperature) before incubation overnight at 4°C with biotinylated antibodies followed by incubation with streptavidin-horseradish peroxidase (GE Healthcare). The protein bands were developed by a chemiluminescent peroxidase substrate (Lumigen from GE Healthcare), and images were acquired by image station 2000R (Kodak).

#### Binding of vaccine protein to MHC class II

Mouse fibroblasts (L cells) stably transfected with MHC class II molecule Eβ^d^Eα^k^ (CA36.2.1) or MHC class I molecule D^d^ (CA25.8.1) [45] were trypsinized and kept in single cell suspension or seeded in chamber slides. To examine binding, supernatants harvested from HEK293 cells transfected with vaccine constructs were added to the cells before incubation at 4°C for 1 h. Next, the bound vaccine molecules were detected by biotinylated anti-human C_H_3 (clone hp6017) followed by fluorochrome-conjugated streptavidin before analysis by fluorescence microscopy or flow cytometry. Non-targeted vaccine (αNIP-gp120) was used as negative control.

#### Chemotaxis assay

600 µl medium (RPMI 1640 with 1% bovine serum albumin) containing recombinant CCL3 (positive control, purchased from R&D Systems, Minneapolis, MN) or the vaccine proteins (supernatants harvested from transfected HEK293) were added to the bottom wells of Corning Transwell plates (5 µm pore size, Sigma). Then, 100 µl of 2 million ESb-MP cells (kindly provided by Dr. J. Van Damme, University of Leuven, Belgium) were added to the upper wells before incubation at 37°C for 2 h. Cells that migrated to the bottom wells were counted manually or by flow cytometry.

### DNA vaccination and electroporation

Plasmids were purified by EndoFree Qiagen kit (Qiagen, Hilden, Germany) and diluted to concentration 0.5 µg/µl in 0.9% NaCl. Before injection of DNA, the mice were anesthetized and their hind legs were shaved. Next, 50 µl DNA-solution (*i.e*. 25 µg DNA) was injected into each quadriceps femoris. When electroporation was included in the immunization protocol, conductive gel (Nihon Kohden, Rosbach, Germany) was applied on the skin before DNA-injection and the injection site was exposed to electroporation immediately after injection. Electroporation was performed by use of the Elgen electroporator device equipped with a caliper electrode (Elgen, Inovio Biomedical Co., PA). The settings were: bipolar pulses of 100 mV×0.2 ms with pulse sequence and pulse sequence train being 1000 and 10, respectively.

### Generation of single cell suspensions of spleen and lymph nodes

Spleen and lymph nodes harvested from mice were crushed by a steel mesh to form a single cell suspension. The suspensions derived from spleen were treated with 140 mM NH_4_Cl in Tris-buffer (pH = 7.2) for 5–10 minutes in order to lyse the erythrocytes.

### Quantification of P18-specific CD8^+^ T cells

H-2D^d^-P18 tetramers folded around the HIV-1IIIB V3 loop P18-epitope (P18: RGPGRAFVTI) [46] were prepared and used to stain P18-specific CD8^+^ T cells from blood essentially as previously described [47] [48]. Mouse blood was collected in RPMI 1640 containing 40 U/ml heparin. Following lysis of red blood cells, remaining cells were incubated with 0.1 µg of PE-labeled D^d^/P18-tetramer in conjunction with anti-CD8α-APC (clone: Ly-2) on ice for 20 min. Next, the cells were washed in PBS containing 2% FCS and fixed in 1.5% paraformaldehyde. Samples were analyzed by two-color flow cytometry on a FACS Calibur (BD Biosciences, San Diego, CA). Gated CD8^+^ T cells were examined for staining with the D^d^-P18 tetramer. CD8^+^ T cells from naïve mice were utilized as negative controls and exhibited less than 0.1% tetramer staining.

### ELISpot assays

Gp120-specific cellular immune responses in vaccinated mice were assessed by IFNγ ELISpot assays. MultiScreenHTS-IP Filter Plates (Millipore) were pre-treated as suggested by the manufacturer which included incubation with 15 µl ethanol per well for 1 min before washing in PBS. The plates were coated with 75 µl of clone AN-18 (10 µg/ml) monoclonal antibodies for detection of IFNγ. After incubation at 4°C overnight, the plates were washed 3 times in 150 µl sterile PBS before adding 1×10^6^, 5×10^5^, and 2.5×10^5^ splenocytes per well in duplicates and that were incubated together with P18 or pooled gp120 derived peptides at 37°C for 26–28 hrs. After washing in PBS (3×150 µl), adherent cells were lyzed by water before one wash with 150 µl PBS containing 0.01% Tween-20 and one wash in PBS. Next, biotinylated anti-IFNγ antibody (75 µl, 1.5 µg/ml, clone XMG1.2, Pharmingen) was added overnight (4°C). Streptavidin conjugated with alkaline phosphatase (1∶3000, Amersham Pharmacia Biotech, Amersham, UK) was added for 1–2 h at room temperature before development of the spots by BCIP/NBT buffer solution (Zymed, Carlsbad, CA). The reaction was stopped by water and the plates were air dried before the number of spots was determined electronically by KS-ELISpot-401 instrument (Zeiss).

### Measurement of gp120-reactive serum antibodies

Serum samples were analyzed by ELISA according to the general protocol described in section 2.4. Recombinant gp120IIIB (1 µg/ml) was added to Nunc MaxiSorp 96 well plates (50 µl/well) and incubated overnight at room temperature. Serum samples were diluted in ELISA-buffer and added to the 96 well plates in dilution 1∶50 followed by 3-fold or 5-fold dilution. As secondary reagents, we used alkaline phosphatase-conjugated anti-murine IgG (Fc-specific, cat. no. A2429, Sigma) or biotinylated IgG subclass-specific antibodies (anti-murine IgG1 (clone 10.9), anti-murine IgG2a (clone 8.3), or anti-murine IgG2b (clone R12-3). Threshold for end point titers was set to 2x the absorbance value of the negative controls (sera from mice given NaCl).

### Neutralization assay

In order to inactivate complement factors, mouse serum samples were incubated in a water bath at 56°C for 30 minutes. Next, the samples were diluted 1/3 in DMEM with 10% FCS and further diluted 3-fold in 96 well plates. To 50 µl of diluted serum samples or controls, 25 µl of live HIV-1 HXB2 was added before the plates were incubated at 37°C for 1 h. Finally, 10 000 Jurkat T cells in 100 µl DMEM with 10% FCS were added per well and the plates were incubated for 6 days at 37°C and 5% CO_2_. Clone P4/D10 was used as positive control [49]. In order to test the ability of the serum samples to inhibit infection, supernatants from the co-culture of diluted serum, HIV-1, and Jurkat T cells were harvested and analyzed for the presence of the HIV-1 antigen Gagp24 in an ELISA.

The ELISA was performed by utilizing rabbit IgG anti-Gagp24 (J. Hinkula, University of Linköping, Sweden) as coat, diluted 1/500 in carbonate buffer (pH = 9.6) (100 µl per well). The plates were stored at 4°C until use, and then washed 4 times in 200 µl PBS with 0.05% Tween-20. All subsequent washing steps were performed in the same manner. Supernatants from the cultures of T cells, serum, and HIV-1 were added to the plates and incubated overnight. Recombinant HIV-1 Gagp24 was utilized as standard. Bound Gagp24 was detected by a monoclonal detection antibody (anti-Gagp24, J. Hinkula) followed by goat anti-mouse IgG-horseradish peroxidase. Both antibodies were incubated for 90 minutes at 37°C. Horseradish peroxidase-substrate was added and the plates were incubated for 30–40 minutes in room temperature before the reaction was aborted by 2.5 mM H_2_SO_4_ and measurement of absorbance at 490 nm.

**Statistical analyses** were performed by use of GraphPad Prism version 6.04. Immune responses among groups of mice are presented as means with SEM, and differences between groups were analyzed by using two-tailed t-tests or one-way ANOVA with the Šidák method for multiple comparisons. p-values<0.05 were considered significant.

## Results

### Generation and functional characterization of the dimeric vaccine molecules

**Figure 1:**
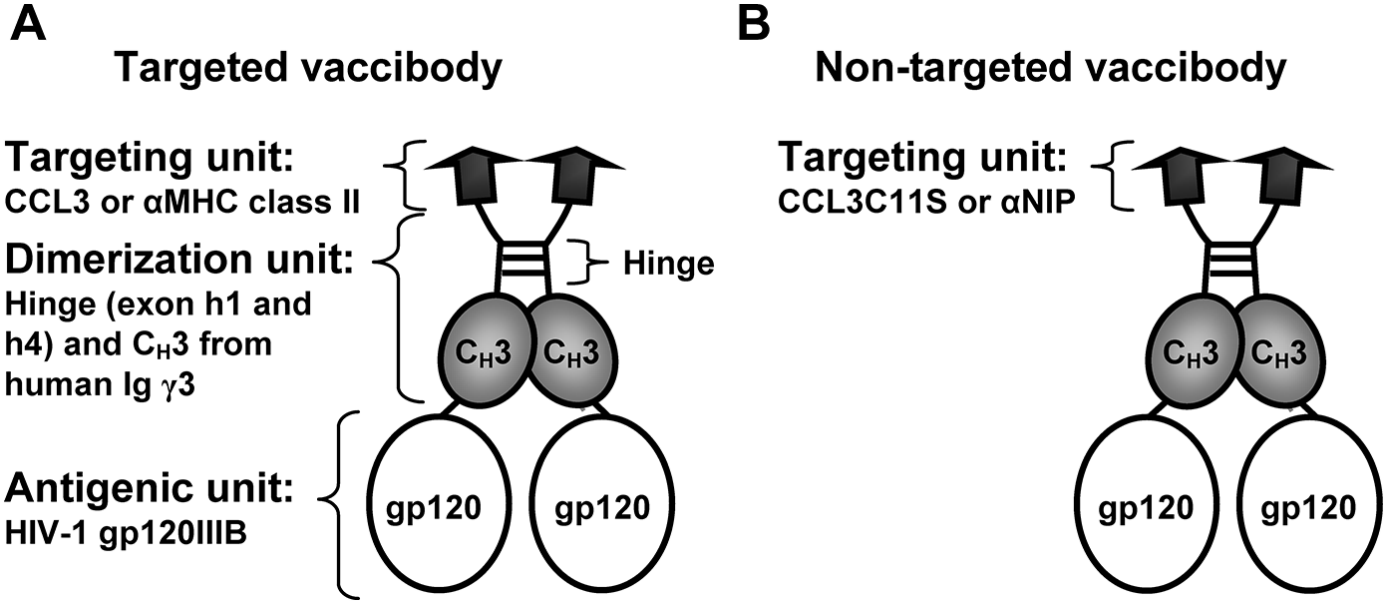
Schematic drawing of gp120-containing vaccibodies. (A) The vaccibody format is a homodimer which consists of three functional units: the targeting, the dimerization, and the antigenic unit. The chemokine CCL3 and the Ig variable regions specific for MHC class II (denoted αMHCII) were utilized to target APCs. The variable regions were assembled in a single chain variable fragment-format (scFv). (B) Vaccibodies without the ability to target APCs, indicated as non-targeted vaccibody, were generated by use of a mutant of CCL3 (CCL3C11S) which does not bind receptor and variable regions specific for the hapten NIP (denoted αNIP). These variable regions were also inserted into the scFv-format.

Targeting of vaccine antigens to specific receptors on APCs may modify and increase the immune response. Thus, in this study, we utilized a targeted, homodimeric vaccine format denoted vaccibody, which physically link APC-specific targeting units via a dimerization unit to antigens [40]. As targeting units we here used (*i*) murine CCL3 that binds to the chemokine receptors CCR1 and CCR5 [50], and (*ii*) the variable regions V_H_+V_L_ from an antibody that binds and is specific for the murine MHC class II molecule I-E^d^ [51]. The variable regions were assembled in a single chain Fragment variable (scFv) format, and this particular targeting unit is hereafter denoted αMHCII (Fig. 1A). A non-targeted control for CCL3 was made by replacing the cysteine at amino acid position 11 with serine (CCL3C11S) which disrupts the three-dimensional structure and thus abrogates binding to chemokine receptors. A non-targeted control for targeting to MHC class II was made from the variable regions of an antibody that recognizes the hapten NIP (5-iodo-4-hydroxy-3-nitrophenacetyl) [52]. These variable regions were also inserted in a scFv-format into the vaccine molecule, and this non-targeting control is hereafter denoted αNIP (Fig. 1B). The dimerization unit consists of a shortened hinge region (exons h1 and h4) joined to the C_H_3 domain from human immunoglobulin γ3 (Fig. 1A). The unit enables interaction between hydrophobic amino acids in the juxtaposed C_H_3 domains, and by the cysteines in the hinge that form covalent disulfide bridges. The C-terminus of the dimerization unit was covalently fused to the N-terminus of HIV-1 envelope antigen gp120 (from clade IIIB) (Fig. 1A).

**Figure 2:**
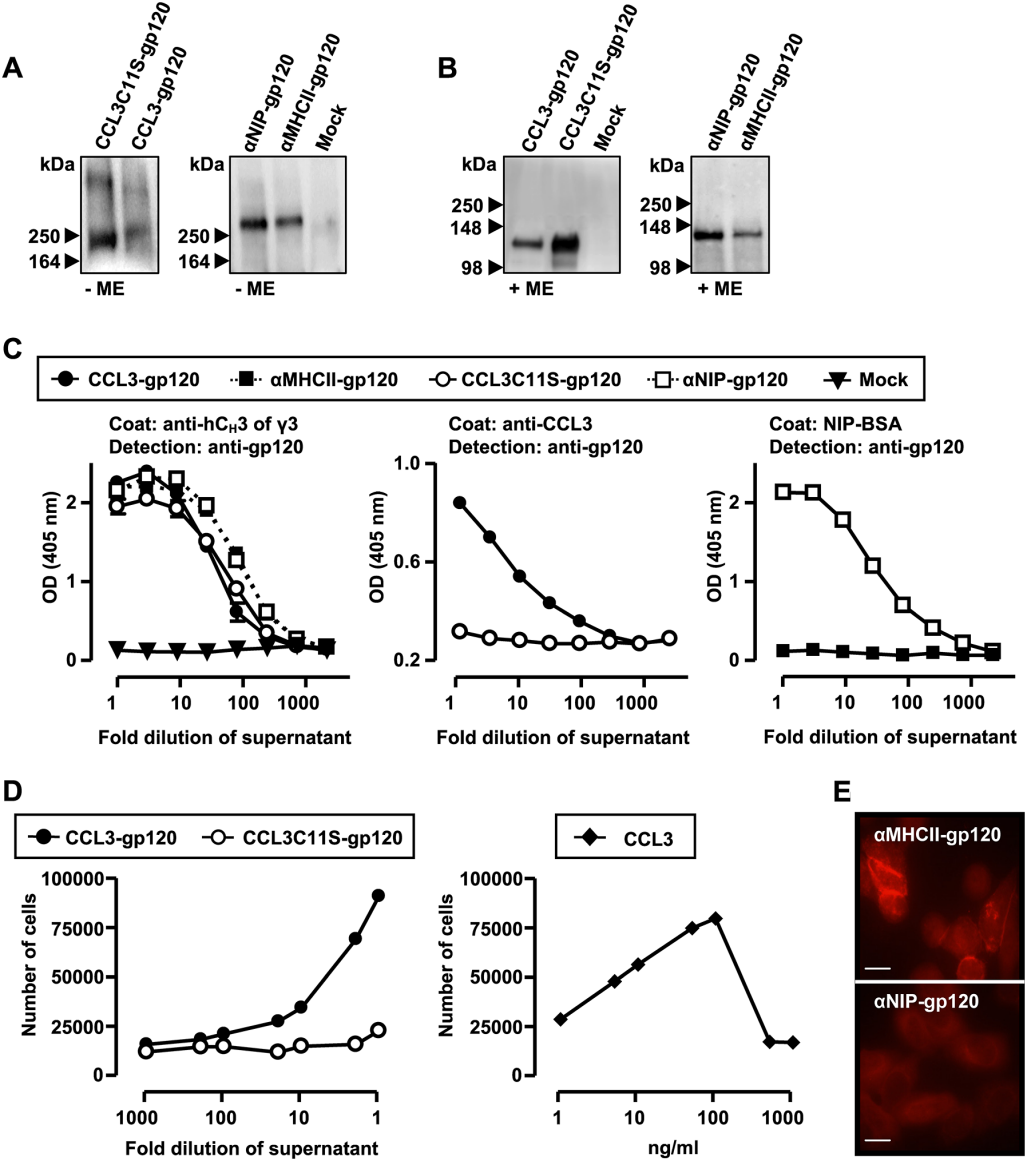
Characterization of the gp120-containing vaccibodies. (A, B) SDS-PAGE (4–20% Tris-Glycine gel) and Western blotting of supernatants harvested from HEK293 cells transfected with DNA encoding the various vaccibodies indicated by their targeting unit. (Mock = supernatant from HEK293 transfected with DNA encoding a fluorescent protein). The supernatants were either left untreated (−ME) (A) or reduced with mercaptoethanol (+ME) (B) prior to SDS-PAGE and blotting. The vaccine proteins were detected by anti-human IgG antibodies (clone HP6017) and chemiluminescence. (C) The gp120-containing vaccibodies were produced and secreted by transfected cells. ELISA of supernatants harvested from HEK293 cells transfected with DNA encoding the vaccine constructs indicated in the figure. The antibodies used as coat and detection in the ELISAs are indicated above each graph. Similar results were obtained in multiple experiments, and one representative experiment is presented. (D) CCL3 inserted into the vaccibody format is chemotactic. Supernatants containing the indicated vaccine proteins (left graph) or recombinant CCL3 (right graph) were added to the bottom wells of Transwell plates. 600 000 Esb-MP cells (a murine T cell line that expresses CCR1 and CCR5) were added to the top wells and the number migrating through the membrane was determined by flow cytometry after 2 h. Representative results from one out of two experiments are shown. (E) αMHCII but not αNIP inserted into the gp120-containing vaccibody binds to MHC class II. Supernatants harvested from transfected HEK293 cells were used to stain MHC class II Eβ^d^Eα^k^-transfected mouse fibroblasts. The bound vaccine proteins were detected by a C_H_3-specific antibody (clone HP6017) and fluorescence microscopy. Scale bar, 10 µm.

In order to characterize the vaccine molecules, DNA was transiently transfected into HEK293 cells and cell media were harvested 3–6 days later for analyses by different assays. SDS-PAGE followed by Western blotting and detection of the vaccine proteins by anti-human IgG antibodies indicated molecular weights (Mw) of approximately 250 kDa for CCL3-gp120 and CCL3C11S-gp120 (Fig. 2A). In addition, a high Mw band was observed for each of the constructs, presumably representing aggregates. Treatment with mercaptoethanol, which reduces disulfide bonds and generates monomers, resulted in dominant bands of approximately 130 kDa for CCL3-gp120 and CCL3C11S-gp120 (Fig. 2B). αMHCII-gp120 and αNIP-gp120 showed Mw slightly above 250 kDa which upon mercaptoethanol treatment was reduced to about 140 kDa (Fig. 2A, B). The results are consistent with predicted Mw, and show that the vaccine molecules could form covalently linked homodimers as expected from their design. Moreover, an ELISA employing antibodies towards the dimerization unit, revealed that the different vaccibodies were secreted to an approximately similar extent (Fig. 2C). A coat antibody towards CCL3 recognized CCL3-gp120 but not CCL3C11S-gp120, suggesting that the C11S mutation changed the confirmation of CCL3 (Fig. 2C). Finally, coating with NIP conjugated to bovine serum albumin, verified that αNIP retained its reactivity when combined with gp120 in the vaccibody format (Fig. 2C). Analyses by Western blotting and the different ELISAs also showed that the three functional units (targeting, dimerization, and antigenic unit) of the vaccibodies were expressed and recognizable by specific antibodies (Fig. 2A–C).

Next, we wanted to verify that the targeting units were functional. By use of a chemotaxis assay employing the CCL3-responding murine T cell lymphoma cell line Esb-MP, we observed that the cells migrated in response to CCL3-gp120 whereas no specific migration was observed towards the non-targeted vaccine CCL3C11S-gp120, confirming the functional activity of the CCL3-moiety (Fig. 2D). Finally, the vaccine protein αMHCII-gp120 could bind to mouse fibroblasts (L cells) transfected with the MHC class II molecule Eβ^d^Eα^k^ (CA36.2.1). In contrast, it did not bind to cells transfected with MHC class I molecule D^d^ (CA25.8.1) and αNIP-gp120 did not bind to MHC class I- nor MHC class II-expressing fibroblasts (Fig. 2E and Fig. S1). These data confirm that the targeting unit αMHCII expressed in a bivalent fusion with gp120, recognized MHC class II in a specific manner.

### Vaccine targeting by CCL3 increased the number of gp120-reactive CD8^+^ T cells

**Figure 3:**
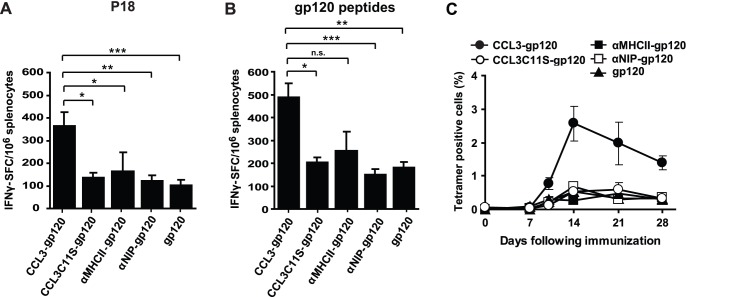
Vaccine with CCL3 inserted in the targeting unit induced higher number of gp120-reactive T cells *in vivo*. BALB/c mice were immunized by one single DNA injection in each quadriceps muscle in the absence of electroporation (25 µg DNA in 50 µl NaCl per quadriceps). (A, B) IFNγ-ELISpot of splenocytes harvested four weeks after immunization with the indicated vaccine constructs. The splenocytes were incubated together with either (A) P18, an MHC class I-binding peptide derived from gp120, or (B) a pool of peptides derived from gp120 (overlapping 15 amino acid peptides). n = 4 mice per group, *0.05>p>0.01; **p = 0.01; ***p<0.01 (analyzed by one-way ANOVA and Šidák method). (C) Kinetics of P18-reactive CD8^+^ T cells in blood. Blood was collected at the indicated time points and P18-reactive CD8^+^ T cells were identified by staining with MHC class I/P18-tetrameric H-2D^d^ complexes and flow cytometry. Percentage tetramer positive cells were calculated from gated CD8^+^ T cells. n = 4 mice per group. In A–C: mean values and SEM are presented.

In order to test the vaccines *in vivo*, the gp120-containing vaccine constructs were injected into the quadriceps of BALB/c mice as plasmid DNA. To evaluate the magnitude of the T cell response four weeks later, splenocytes were stimulated with either the peptide P18-I10 (RGPGRAFVTI) which binds to MHC class I (Fig. 3A), or a pool of peptides covering the entire gp120 and consisting of 15 amino acid long peptides with overlapping sequences (Fig. 3B). Because of their length, the pool of gp120 peptides are expected to bind MHC class II molecules and be indicative of CD4^+^ T cell responses, but CD8^+^ T cell responses cannot be excluded. Targeting of gp120 by CCL3 increased the mean number of P18-reactive IFNγ-producing T cells more than 2-fold compared to the other vaccine constructs (Fig. 3A). Targeting by CCL3 also increased the number of gp120 peptide-reactive IFNγ-producing T cells more than 2-fold compared to either the non-targeted control constructs or gp120 delivered alone (Fig. 3B). Analysis of blood by use of MHC class I/P18-tetramer complexes revealed similar results to those we observed in spleen. The mice that were immunized with CCL3-gp120 showed 3 to 7-fold more P18-reactive CD8^+^ T cells compared to the mice that were immunized with non-targeted vaccibodies or gp120 alone (day 10 to day 28) (Fig. 3C).

### CCL3 inserted in the targeting unit partly overcomes the need of electroporation

**Figure 4:**
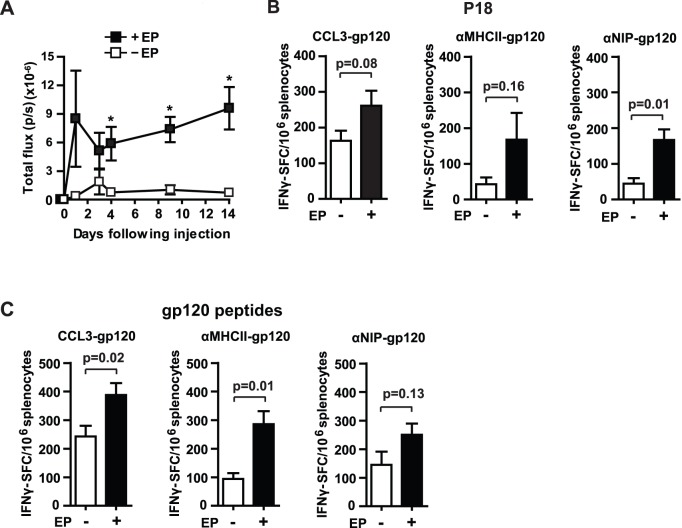
Effects of electroporation and targeting molecules on the cellular immune response. Immunizations of BALB/c mice (25 µg DNA in 50 µl NaCl per quadriceps) were performed without (−EP) or with electroporation (+EP). (A) Electroporation increased the amount of luciferase at the injection site. DNA encoding luciferase was delivered intramuscularly in the quadriceps without (−EP) or with electroporation (+EP), and the expression of luciferase was measured by *in vivo* imaging and bioluminescence (p/s: photons/second) at different time points after injection. The amount of luciferase differed significantly between the two groups from day 4, *p≤0.02, n = 3–4 muscles/group. (B, C) Cellular immune responses induced by the vaccibodies delivered by intramuscular DNA-injection without (−EP) or with electroporation (+EP). Splenocytes were harvested four weeks after immunization and incubated together with the MHC class I, P18-peptide (B) or a pool of overlapping peptides from gp120 (C) in IFNγ-ELISpot assay. n = 8 mice per group for CCL3-gp120 and n = 4 mice per group for αMHCII-gp120 and αNIP-gp120. In A–C: mean values and SEM are presented. B and C, for the groups of control mice (NaCl delivered with or without EP, and re-stimulated with P18 or gp120 peptides) the mean number of IFNγ-SFC/10^6^ splenocytes varied from 37 to 47 and is substracted from the number of spots in the respective samples from vaccibody-immunized mice.

Electroporation facilitates cellular uptake of DNA by induction of reversible pores in the cell membrane. Thereby, electroporation may increase the level of expressed protein after DNA vaccination [9]. Presence of more vaccine protein may enhance antigen loading of APCs and the magnitude of the immune response. To confirm that our electroporation protocol could increase the amount of vaccine protein, we injected a firefly luciferase-encoding DNA plasmid into quadriceps muscles, before treating half of the mice with electroporation (Fig. 4A). Expression of luciferase was determined by *in vivo* imaging of bioluminescence. From day 4 after injection, we observed significantly increased amounts of luciferase activity when electroporation was included (Fig. 4A). Next, to evaluate whether electroporation increased the magnitude of the T cell response, we enumerated P18-specific as well as gp120-reactive splenic T cells by ELISpot 4 weeks after immunization. Immunization was performed as previously described: DNA plasmids encoding vaccibodies were injected into quadriceps of BALB/c and the muscles were left untreated or exposed to electroporation. Electroporation significantly increased the number of P18-reactive, IFNγ-producing T cells induced by αNIP-gp120 but not CCL3-gp120 nor αMHCII-gp120 (Fig. 4B). The opposite pattern was observed for the IFNγ-positive T cell response towards the pool of gp120 peptides. Electroporation resulted in significant increase of these cells following immunization with CCL3-gp120 or αMHCII-gp120 but not αNIP-gp120 (Fig. 4C). Taken together, these data support the results presented in Figure 3, and suggest that CCL3 can be utilized as targeting to promote CD8^+^ T cell responses even when electroporation is not included. Electroporation was beneficial for the number of gp120 peptide pool-reactive T cells, indicative of CD4^+^ T cells. However, CCL3-gp120 also induced T cells reactive to the gp120 peptide pool in the absence of electroporation.

### Induction of humoral immune responses

**Figure 5:**
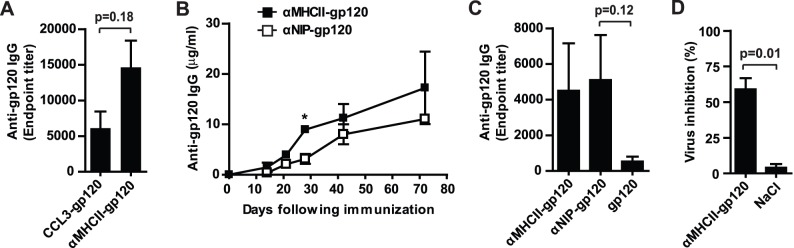
Vaccibody induced long-lasting gp120-reactive serum antibodies which can neutralize HIV-1. BALB/c mice were immunized once with DNA encoding the indicated vaccine constructs. DNA (25 µg DNA diluted in 50 µl NaCl) was injected into the quadriceps before electroporation. (A) Levels of gp120-reactive IgG-antibodies in mice sera harvested 56 days after immunization with the indicated vaccibodies. The levels are indicated by endpoint titers and were determined by ELISA using gp120 as coat. n = 5 mice/group. (B) Effects of targeting on the level of gp120-reactive IgG-antibodies in sera. Sera were harvested at the indicated time points, and the amounts of gp120-reactive antibodies were determined by ELISA using gp120 as coat. Anti-gp120 IgG levels differed significantly between the two vaccine groups at day 28, *p = 0.003, n = 3 to 4 mice per group. The experiment was performed twice and showed similar results. (C) Titers of gp120-reactive IgG-serum antibodies 10 months after immunization with DNA (2 µg diluted in 50 µl NaCl) encoding vaccibodies or gp120. Sera were analyzed by ELISA using gp120 as coat. n = 4 mice per group. (D) αMHCII-gp120 induced antibodies that neutralized HIV-1. Sera harvested 10 or 15 months after injection of DNA or NaCl were incubated together with Jurkat T cells and HIV-1. A murine IgG1 antibody (clone P4/D10) that can neutralize HIV-1IIIB through recognition of amino acid 304–323 of gp120 was used as positive control [49], and its inhibition of HIV-1 infection was set to 100%. Percentage inhibition by the mice sera is relative to the positive control. n = 4 mice per group. In A–D: mean values and SEM are presented.

An efficient prophylactic vaccine towards HIV-1 would probably depend on high antibody titers that are durable and that can neutralize a large number of HIV-1 variants [53]. To examine whether gp120-containing vaccibodies induced gp120-reactive antibodies, BALB/c mice were immunized with DNA as previously described. In initial experiments, we delivered the DNA vaccines without electroporation, but only low levels of serum gp120-reactive antibodies were detected (Fig. S2). Therefore, for this part of the study, we decided to include electroporation in the immunization protocol. We harvested sera 56 days after immunization, and found that both CCL3-gp120 and αMHCII-gp120 induced gp120-reactive antibodies (Fig. 5A). Next, we asked the question whether targeting could increase the level of gp120-reactive antibodies. Serum antibody responses following immunization with αMHCII-gp120 or the non-targeted control αNIP-gp120, showed a significant effect of targeting after 28 days (Fig. 5B). However, at earlier and later time points the amount of serum gp120-reactive antibodies overlapped in the two vaccine groups.

In combination with hemagglutinin from influenza or ovalbumin, targeting by use of αMHCII generated higher antibody levels than targeting by CCL3 [41]. Based on this knowledge and our findings (Fig. 5A and data not shown), we chose αMHCII as targeting unit for the next experiments. First, we examined whether targeting was important for long-lasting antibody responses. Because lower amounts of DNA may better reveal effects of targeting, we included groups of mice that were immunized with 2 µg DNA diluted in 50 µl NaCl per quadriceps (Fig. 5C). However, 10 months after one single immunization, αMHCII-gp120 and αNIP-gp120 showed similar titers of gp120-reactive antibodies in serum (Fig. 5C). More mice in the groups receiving αMHCII-gp120 or αNIP-gp120 achieved high antibody titers than those receiving gp120 only, but the difference was not significant (Fig. 5C). Finally, sera from immunized mice were tested for their ability to neutralize HIV-1 in an *in vitro* neutralization assay in which mice sera were added to Jurkat T cells before incubation with HIV-1IIIB. Serum harvested 10 or 15 months after one single immunization with αMHCII-gp120 showed virus inhibition which differed significantly from the background (sera from mice given NaCl) (Fig. 5D). Based on these experiments, we conclude that gp120 inserted into the vaccine format can elicit gp120-reactive antibodies that are long-lasting. Our analyses did not reveal an obvious effect of targeting. Instead our data raise the question whether bivalency of gp120 could be beneficial for the response.

## Discussion

In the present study, we show that a homodimeric vaccine utilizing CCL3 as targeting unit, induced a higher number of gp120-reactive CD8^+^ T cells than the non-targeted vaccine or gp120 alone. CD8^+^ T cell responses could be induced to a similar level using CCL3 targeting without electroporation as a non-targeted version with electroporation, suggesting that CCL3 could be used as an immune adjuvant to enhance the potency of DNA vaccines overcoming the need for electroporation. The homodimeric vaccine also induced gp120-reactive antibodies, however, targeting did not increase the antibody titers.

Various molecules have been tested for their efficacy to target vaccine antigens towards APCs [13]. The needs and requirements for improvement of the immune response towards a vaccine antigen will not only depend on the pathogen, but also the intrinsic immune properties of the utilized subunit, the vaccine formula, and the site of vaccine delivery. The cytokine GM-CSF has been included in many clinical vaccine trials in humans (listed at clinicaltrials.gov). However, when aiming to increase CD8^+^ T cell responses, we chose to use CCL3 for several reasons. First, targeting by CCL3 has been shown to induce protection against tumor development in mouse models, and CD8^+^ T cells were crucial for the protection [24]. Targeting by CCL3 also increased the number of influenza hemagglutinin-reactive CD8^+^ T cells [41]. Second, CCL3 may induce production of IL-12 in DCs and enhance IFNγ-production in T cells which is essential for development of Th1 immunity [54], [55]. Third, we wanted to deliver the vaccine intramuscularly. There are numerous APCs in different layers of the skin [56], but few APCs are present in muscular tissue [57]. By use of CCL3, APCs could be recruited to the injection site. Supporting this view, injection of DNA encoding CCL3 into mice muscle was shown to recruit APCs [33], [34], and 75% of the cellular infiltrate consisted of DCs [33] which are crucial for transport of antigen to lymph nodes and subsequent priming of T cells. In contrast, GM-CSF recruited mainly macrophages and few DCs [33], and was shown to have no effect or even an adverse effect on the number of vaccine-induced HIV-1 gp120-reactive CD8^+^ T cells when co-delivered with DNA encoding HIV-1 gp120 into muscle of mice [58]. Because high level of antibodies is a desired goal for an HIV-1 vaccine, we also targeted gp120 to MHC class II molecules. Several studies have reported that targeting of antigen to MHC class II can increase the antibody levels, and also generate protection against disease [22], [27], [40], [41], [59], [60].

The various gp120-containing vaccibodies induced different magnitudes of HIV-1 gp120-reactive immune responses. *In vitro*-experiments and analyses by ELISA showed similar secretion levels of the vaccibodies, suggesting that the differences in immune response magnitude were caused by the targeting moieties. In all experiments examining T cell responses, there was a tendency of higher T cell numbers following targeting by CCL3 compared to the other vaccine strategies. The most prominent effect of CCL3 was observed when immunization was performed without electroporation. Based on these data, we suggest that a smaller amount of the vaccine construct is needed when CCL3 is used as targeting unit, compared to anti-MHC class II or gp120 alone in order to obtain the same magnitude of the response.

The finding that CCL3 was a more efficient T cell inducer than anti-MHC class II or the non-targeted vaccines, may be explained by different mechanisms that are not mutually exclusive: *i*) CCL3 is a chemokine which can recruit APCs to the injection site, but anti-MHC class II has no such ability *per se*. Electroporation typically activates pro-inflammatory cytokine genes and stress genes [61], [62] which can lead to activation and recruitment of APCs [61]. Thus, the importance of electroporation when anti-MHC class II was utilized, indicate that an inflammatory environment with recruitment of cells to the injection site is important for the immune response. The potency of CCL3 even without electroporation, indicate that low amounts of CCL3 is sufficient for recruitment of cells crucial for the immune response. *ii*) In our study, the magnitudes of T cells were determined by IFNγ-measurements and tetramers, indicative of Th1 and CD8^+^ T cell responses. CCR5, a receptor of CCL3 provides signal for induced production of IL-12 which selectively directs development of a Th1 response [54], and is consistent with the finding that CCL3 can enhance IFNγ-production in T cells [55]. *iii*) The two targeting molecules bind to receptors present on different types of cells, and the type of cell that is engaged can be crucial for the outcome of the response. *iv*) The cellular uptake and intracellular fate is expected to differ for the various vaccines. The number of surface receptors, the signalling they mediate, their rate of internalization, and intracellular trafficking will influence on antigen loading, the peptide repertoire that is generated, and loading onto MHC class I and II molecules. *v*) CCL3 can bind extracellular matrix molecules such as glycosaminoglycans. This could protect the vaccine protein against proteases and rapid turnover. Moreover, CCL3 may help recruit APCs close to the vaccine antigen, and thereby reduce the amount of vaccine that is needed.

There is lots of focus on how vaccines can be designed to promote cross-presentation and subsequent priming of CD8^+^ T cells [63]. In this regard, our vaccine targeted by CCL3 induced significant CD8^+^ T cell responses. Because gp120 can bind to receptors able to mediate cross-presentation [64], and because it can not be excluded that immunization lead to some cellular death, additional experiments would be needed to conclude that CCL3 transfers extracellular vaccine antigen into the pathway of cross-presentation. But there are reasons to believe that chemokines can mediate cross-presentation. A subpopulation of CD11c^+^ DCs in mice, the CD8^+^ DCs, are widely thought to exhibit an enhanced capacity for cross-presentation [65], and data by Alberti *et al*., suggest that CCR5 is expressed by these cells [54]. Other studies claim that multiple DC populations can present exogenous antigens to CD8^+^ T cells given that the antigen is delivered to early endocytic compartments and is protected from lysosomal degradation [66], [67]. CCR1 and CCR5 are both internalized, but trafficking of CCR5 is most thoroughly studied. After interaction with its natural ligand, CCR5 can accumulate in recycling endosomes before it recycles back to the plasma membrane, where it becomes functional for a new round of stimulation [68]. Therefore, CCR5 may have the potential to rescue antigens from lysosomal degradation, and promote cross-presentation independently of CD8^+^ DCs. It should also be mentioned that targeting to the chemokine receptor CCR6 by use of CCL20 fused to gp100 resulted in cross-presentation and activation of CD8^+^ T cells in a process that was transporter associated with antigen processing (TAP)-dependent [25].

In this study, we observed a significant targeting effect on the magnitude of antibody responses only at early time points after immunization. This is in contrast to other studies utilizing the vaccibody. MHC class II-targeting vaccibodies containing either the influenza virus antigen hemagglutinin [27], [41], or B-cell lymphoma or myeloma Ig-derived idiotype antigens [40], were shown to induce higher levels of antigen-reactive antibodies compared to non-targeted delivery. However, although not significant, none of the mice immunized with gp120 alone showed as high durable endpoint titers as those that received gp120-containing vaccibodies. We therefore wondered whether bivalency of gp120 in the targeted and non-targeted vaccibody could promote antibody responses. Bivalency of antigen could promote crosslinking of B cell receptors which has been suggested to enhance B cell stimulation for soluble antigen [69]. It must be noted though, that the dimerization unit of the vaccibody is of human origin and may be immunogenic in mice [24], which can not be excluded as a reason for the observation in our study.

Several studies have examined the potency of CCL3, but few have fused it to vaccine antigens. To our knowledge, there are no data supporting that CD8^+^ T cells against gp120 are crucial in elimination of HIV-1-infected cells. However, CCL3 may be combined with HIV-1 antigens more relevant for a CD8^+^ T cell response. Chemokines are key players in immune responses following natural infections, and the chemokine - chemokine receptor system is well conserved between species. Thus, we find chemokines to be promising candidates as immune adjuvants in vaccines, in particular when vaccines are delivered at sites where few APCs are present.

## Supporting Information

Figure S1 **αMHCII-gp120 bound specifically to MHC class II-expressing cells.** Supernatant from HEK293 transfected with the indicated vaccibodies were incubated with MHC class II-positive or negative fibroblasts for 1 h at 4°C. Bound vaccine molecules were detected by biotinylated anti-human C_H_3 (clone HP6017) and streptavidin-PE before analysis by flow cytometry. αMHCII-gp120 bound to MHC class II-positive cells, but not to the negative cells. The non-targeted control, αNIP-gp120, did not show significant binding to any of the cells.
(DOCX)

Figure S2 **Levels of anti-gp120 IgG in sera.** BALB/c mice were immunized once with DNA encoding the indicated vaccibodies. DNA (25 µg DNA diluted in 50 µl NaCl) was injected into the quadriceps with electroporation (+EP) or without. Levels (endpoint titers) of HIV-1 gp120-reactive IgG-antibodies in mice sera were determined at day 28 after immunization by ELISA utilizing gp120 as coat. Mean values and SEM are presented, n = 4 mice/group.
(EPS)
