# Supplementary material for: Increased Generation of HIV-1 gp120-Reactive CD8+ T Cells by a DNA Vaccine Construct Encoding the Chemokine CCL3
Source: PLoS One. 2014 Aug 14;9(8):e104814. doi: 10.1371/journal.pone.0104814 (PMC4133255; doi:10.1371/journal.pone.0104814)
Supplement: Supplementary file 1 [file pone.0104814.s001.docx]

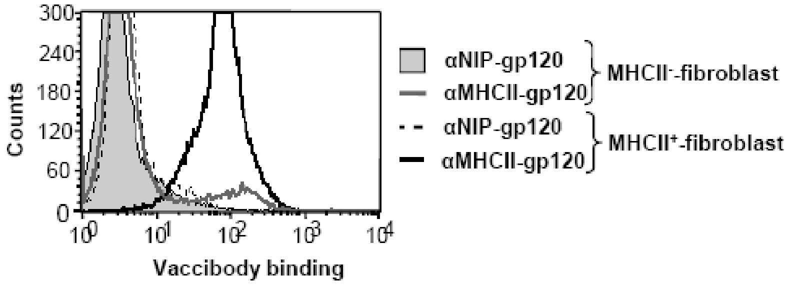

300
240
180
120
60
0
Counts
10^0
10^1
10^2
10^3
10^4
Vaccibody binding
αNIP-gp120
αMHCII-gp120
MHCII⁻-fibroblast
αNIP-gp120
αMHCII-gp120
MHCII⁺-fibroblast
